# Comparison of the efficacy of Philos plate and Multiloc intramedullary nail in the treatment of complex proximal humeral fractures with osteoporosis in the elderly

**DOI:** 10.3389/fsurg.2025.1606898

**Published:** 2025-06-18

**Authors:** Qian Liu, Zongbing Cao, Jing Liu, Zhiyong Liu, Wenbo Zhang

**Affiliations:** ^1^ Department of Orthopedic, Qilu Hospital Dezhou Hospital of Shandong University Dezhou Hospital, Dezhou, Shandong, China; ^2^ Department of Endocrinology, Qilu Hospital Dezhou Hospital of Shandong University Dezhou Hospital, Dezhou, Shandong, China; ^3^ Health Management Center, Qilu Hospital Dezhou Hospital of Shandong University Dezhou Hospital, Dezhou, Shandong, China

**Keywords:** proximal humerus fracture, PHILOS plate, MultiLoc nail, osteoporosis, functional recovery, fluoroscopy

## Abstract

**Introduction:**

Proximal humeral fractures are common in elderly patients with osteoporosis. Complex three- or four-part fractures often require surgical intervention. Philos locking plates and Multiloc intramedullary nails are widely used, but their comparative effectiveness in osteoporotic elderly patients remains uncertain.

**Methods:**

A retrospective study was performed on 90 elderly patients (aged 70–95 years) with Neer three- or four-part proximal humeral fractures treated between January 2021 and December 2023. Patients received either Philos plate fixation (*n* = 50) or Multiloc intramedullary nail fixation (*n* = 40). Clinical data included incision length, operative time, blood loss, complications, and functional outcomes. Pain was assessed via VAS, and shoulder function via Constant-Murley scores at 1 week, 1 month, and 12 months postoperatively.

**Results:**

Both groups achieved fracture healing and functional improvement. Compared to the Philos group, the Multiloc group had shorter incisions, less blood loss, and shorter operative time (all *P* < 0.05). VAS scores were lower and Constant-Murley scores higher in the Multiloc group at all time points (*P* < 0.05). Complication rates were lower in the Multiloc group (10% vs. 20%).

**Discussion:**

Both techniques are effective, but Multiloc intramedullary nail fixation offers superior early outcomes and fewer complications. It may be preferable for elderly patients with osteoporotic proximal humeral fractures when proper surgical technique is ensured.

## 1 Introduction

Proximal humeral fractures are a common type of fracture, accounting for approximately 3%–5% of all fractures (1). Typically caused by external forces, they present with shoulder swelling, pain, and limited mobility, impairing limb function and affecting daily activities (2–3). Conservative treatment is effective for minimally displaced proximal humeral fractures; however, unstable or significantly displaced fractures often require surgical intervention to achieve anatomical reduction, stable fixation, and early mobilization (4, 5). In older patients with osteoporosis, complex proximal humeral fractures involve multiple displacement sites and pose clinically severe injuries. These fractures necessitate timely surgical intervention to restore local anatomy and accelerate fracture healing (6, 7). Philos plate and Multiloc intramedullary nail fixation are commonly used surgical methods for treating proximal humeral fractures, each with distinct characteristics. However, the optimal surgical technique for managing osteoporotic complex fractures remains debated (8–10). This study retrospectively compared the clinical efficacy of Philos plate and Multiloc intramedullary nail fixation in older patients with osteoporotic complex proximal humeral fractures, providing a reference for surgical management.

## 2 Materials and methods

### 2.1 Inclusion and exclusion criteria

Patients with radiographic and CT confirmation of a fresh, significantly displaced proximal humeral fractures, classified as Neer three- or four-part fractures, were eligible for inclusion. Preoperative CT scans were routinely performed to evaluate fracture complexity, humeral head position, medial metaphyseal support, and bone loss beneath the humeral head, thereby guiding surgical decision-making. Additional criteria included the absence of significant symptoms of nerve injury, an age range of 70–95 years, treatment with either Philos plate or Multiloc intramedullary nail fixation, and the availability of complete clinical follow-up data.

Exclusion criteria encompassed a history of restricted shoulder joint mobility, the presence of preoperative acute or chronic infections, and severe comorbid internal diseases. Patients with psychiatric disorders and incomplete follow-up data were also excluded.

### 2.2 Study setting and ethical approval

This retrospective study was conducted at the Department of Orthopedic, Qilu Hospital Dezhou Hospital of Shandong University, Dezhou, China. Patients treated between January 2021 and December 2023 were reviewed. The study protocol was reviewed and approved by the Ethics Committee of Qilu Hospital Dezhou Hospital of Shandong University (2025070). Written informed consent was obtained from all participants prior to data collection and analysis.

### 2.3 Surgical methods

Both groups underwent surgery under general anesthesia combined with a brachial plexus block. The patients were positioned in a beach chair position, with a soft cushion placed behind the surgical shoulder to ensure sufficient passive flexion and extension of the affected limb during the procedure.

#### 2.3.1 Philos plate group

The deltopectoral approach was employed for the Philos plate group. The cephalic vein was exposed and protected, with the pectoralis major retracted medially and the deltoid laterally to assess the fracture site and evaluate the integrity of the rotator cuff. Hematomas and embedded soft tissues surrounding the fracture were cleared. Fracture reduction is achieved through traction of the forearm, bone leverage, or Kirschner wire assistance. Once satisfactory reduction was confirmed under fluoroscopy, temporary fixation was secured with Kirschner wires. The Philos locking plate was positioned approximately 0.5 cm below the greater tuberosity and 1 cm posterior to the bicipital groove. Distal locking screws were inserted, and the positioning of the screws, plate, and fracture reduction were verified under fluoroscopy. In the presence of rotator cuff damage, it was reinforced intraoperatively using nonabsorbable sutures through the locking holes of the plate. The wound was irrigated with saline, closed in layers, and covered with sterile gauze.

#### 2.3.2 Multiloc intramedullary nail group

In the Multiloc group, a longitudinal incision of approximately 3–5 cm was made along the anterolateral acromion. A layer-by-layer dissection of the skin, subcutaneous tissue, and deltoid muscle was performed while protecting the rotator cuff and neurovascular bundles. Exposure to the greater tuberosity of the humerus allowed for the reduction of the fracture ends, which was temporarily fixed using Kirschner wires under fluoroscopic guidance. A guidewire was inserted through the greater tuberosity into the medullary canal, followed by sequential reaming under C-arm fluoroscopy. A Multiloc intramedullary nail of suitable length was inserted into the distal end of the fracture. The position of the nail was confirmed via intraoperative fluoroscopy, after which proximal and distal locking screws were inserted using a targeting device. The multiplanar angles of the locking screws were adjusted to ensure reliable fixation of both the humeral head and distal fracture end. After confirming satisfactory alignment and secure fixation, the surgical site was thoroughly irrigated. The deltoid muscle, subcutaneous tissue, and skin were closed in layers, and the incision was covered with a sterile dressing.

### 2.4 Postoperative management

All patients had their affected limbs suspended and immobilized for 6 weeks postoperatively. On the second day post-surgery, patients commenced active and passive functional exercises targeting the elbow, wrist, and finger joints of the affected limb. Shoulder shrugging, pendulum exercises, active/passive forward flexion, and elevation of the affected shoulder were also incorporated. The intensity of the exercises was tailored to maintain mild discomfort, with a visual analog scale (VAS) score of 2–3.

In the PHILOS plate group, excessive shoulder movements—such as abduction, external rotation, and extension—were restricted during the initial 6 weeks postoperatively. Following this period, the forearm sling was removed, and patients began active and passive range-of-motion exercises in all directions. At 12 weeks, progressive resistance and weight-bearing exercises were initiated as tolerated. In contrast, patients in the Multiloc intramedullary nail group were typically permitted—and actively encouraged—to initiate active-assisted and passive shoulder mobilization at an earlier stage. This early rehabilitation strategy was tailored according to the intraoperative assessment of fixation stability and the patient's postoperative comfort, leveraging the superior biomechanical support provided by the intramedullary construct. Particular attention was given to patients with diabetes or other comorbidities associated with a higher risk of joint stiffness. These individuals were closely monitored and received early, supervised physiotherapy to minimize the risk of adhesive capsulitis and to preserve shoulder mobility throughout the recovery period.

### 2.5 Follow-up and evaluation indicators

The surgical incision length, operative time, intraoperative blood loss, postoperative complications, and fracture healing status were recorded and compared between the two groups. Postoperative pain was assessed using the VAS at one week, one month, and 12 months post-surgery. Shoulder joint function was evaluated using the Constant-Murley score.

### 2.6 Statistical methods

Data in this study were analyzed using IBM SPSS Statistics version 25.0. The Shapiro–Wilk test was applied to assess the normality of the data distribution, while the Levene test was used to evaluate the homogeneity of variance.

For data conforming to a normal distribution and homogenous variance, results are expressed as mean ± standard deviation (X ± S). For non-normally distributed data, results are presented as median (interquartile range) [M (Q1, Q3)]. Continuous variables were compared between the two groups using an independent sample *T*-test, and the *χ*^2^ test was employed for comparisons of categorical variables. For comparisons of repeated measures within the same group at different time points, the *F*-test was applied. A *P*-value of <0.05 was considered statistically significant.

## 3 Results

### 3.1 General clinical data

A total of 90 patients with complex proximal humeral fractures treated between January 2021 and December 2023 were included based on the inclusion and exclusion criteria. Patients were categorized into two groups according to the surgical method: the Philos plate (*n* = 50) and Multiloc intramedullary nail groups (*n* = 40). No significant differences existed in baseline characteristics, including sex, age, fracture classification, fracture type, mechanism of injury, and comorbidities between the two groups (*P* > 0.05), indicating comparability (Table 1). This study protocol was approved by the Ethics Committee of our institution, and informed consent was obtained from all enrolled patients.

**Table 1:** Comparison of baseline characteristics between the Two groups.

Group	Male/Female	Age (years) (mean ± SD)	Classification (cases)		Neer fracture type (cases)		Mechanism of injury		Comorbidities	
			Abduction	Adduction	3-Part fracture	4-Part fracture	Fall injury	Traffic injury	Yes	No
PHILOS Group	22/28	81.52 ± 8.83	30	20	27	23	30	20	33	17
Multiloc Group	19/21	82.76 ± 8.35	28	12	20	20	24	16	27	13
*χ*^2^*/T-value	0.05	−0.65	0.225*		0.001*		0.028*		0.034*	
*P*-value	0.822	0.521	0.636		0.935		0.835		0.782	

* Indicates that the value represents a T-value rather than a chi-square statistic.

### 3.2 Comparison of surgical metrics

The Multiloc intramedullary nail group exhibited a significantly shorter surgical incision length compared to the PHILOS plate group (6.0 ± 0.8 cm vs. 13.5 ± 1.2 cm, *P* < 0.05). Intraoperative blood loss was also significantly lower in the Multiloc group than in the PHILOS group (150 ± 40 ml vs. 250 ± 50 ml, *P* < 0.05). Furthermore, the operative time in the Multiloc group was significantly shorter than that in the PHILOS group (63.25 ± 8.76 min vs. 95.89 ± 8.73 min, *P* < 0.05), as demonstrated in Table 2.

**Table 2:** Comparison of surgical metrics between the Two groups.

Group	Operative time (min)	Incision length (cm)	Intraoperative blood loss (ml)
PHILOS Group	95.89 ± 8.73	13.55 ± 1.27	250.29 ± 53.79
Multiloc Group	63.25 ± 8.76	6.20 ± 0.84	150.82 ± 47.35
T-Value	16.69	30.53	8.79
*P*-Value	0.007	0.001	0.013

### 3.3 Comparison of VAS scores at different time points

Postoperative VAS scores decreased over time in both groups with no significant differences between them. However, at one day, three days, and one week postoperatively, the PHILOS plate group exhibited significantly higher VAS scores than the Multiloc group (*P* < 0.05). No significant differences were observed between the two groups at two weeks and one month postoperatively (*P* > 0.05) (Table 3).

**Table 3:** Comparison of VAS scores at different time points between the Two groups.

Group	Preoperative	Postoperative 1 Day	3 Days	1 Week	2 Weeks	1 Month	*F*-Value	*P*-Value
PHILOS Group	6.36 ± 1.21	8.32 ± 1.08	6.35 ± 0.59	5.18 ± 0.47	2.75 ± 0.41	2.12 ± 0.37	9.431	0.026
Multiloc Group	6.53 ± 1.57	7.21 ± 0.95	4.73 ± 0.83	4.18 ± 0.52	2.86 ± 0.27	2.03 ± 0.32	7.559	0.031
*T*-Value	1.225	5.225	5.752	7.398	3.338	2.275	–	–
*P*-Value	0.561	0.043	0.043	0.025	0.435	0.597	–	–

### 3.4 Comparison of constant-murley shoulder function scores

Constant-Murley shoulder function scores demonstrated a significant improvement over time in both groups (*P* < 0.05). No significant differences existed in preoperative scores between the two groups (*P* > 0.05). However, at 1, 3, 6, and 12 months postoperatively, the MultiLoc group demonstrated significantly higher shoulder function scores than the PHILOS group (*P* < 0.05), as shown in Table 4.

**Table 4:** Comparison of constant-murley scores at different time points between the Two groups.

Group	Preoperative	1 Month Postoperative	3 Months	6 Months	12 Months	*F*-value	*P*-value
PHILOS Group	23.25 ± 2.57	40.36 ± 9.29	46.77 ± 12.39	59.47 ± 10.71	72.83 ± 8.87	12.821	0.021
Multiloc Group	22.37 ± 1.95	45.94 ± 14.26	51.64 ± 8.96	68.26 ± 9.71	80.62 ± 9.73	8.251	0.036
*T*-Value	0.259	6.852	7.248	7.398	6.338	–	–
*P*-Value	0.682	0.032	0.021	0.020	0.037	–	–

### 3.5 Comparison of postoperative complications

The incidence of postoperative complications was significantly higher in the PHILOS plate group than in the Multiloc group. In the PHILOS group, complications occurred in 20% of cases, including three screw cut-outs, three humeral head varus necroses, and two humeral head malunion. Conversely, the MultiLoc group had a 10% complication rate, with two cases of locking screws loosening and two peri-implant fractures. The difference between the two groups was statistically significant (*χ*^2^ = 7.231, *P* = 0.025).

### 3.6 Radiographic evaluation indicators

Radiographic assessment at follow-up showed that all patients achieved clinical and radiographic union within the observation period. The mean time to fracture union was 14.2 ± 2.1 weeks in the Multiloc group and 16.8 ± 2.6 weeks in the PHILOS group (*P* < 0.05). The fracture union rate at 3 months postoperatively was 92.5% (37/40) in the Multiloc group and 84.0% (42/50) in the PHILOS group. The remaining cases achieved union by the 6-month follow-up. No cases of nonunion were observed in either group during the 12-month follow-up period.

## 4 Discussion

This retrospective study analyzed and compared the efficacy and safety of Philos plate fixation and Multiloc intramedullary nail fixation for treating complex osteoporotic proximal humeral fractures in plder patients. While both methods effectively facilitated fracture healing and restored shoulder function, they differed significantly in postoperative functional recovery, intraoperative characteristics, and complication rates.

The Philos plate, a locking plate with multiple screw holes and an angular locking design, provides excellent biomechanical stability. Compared with traditional plates, it reduces soft tissue irritation and periosteal stripping, thereby preserving the vascular supply to the proximal humerus, thus making it particularly effective in managing osteoporotic fractures (11–13). However, findings from this study indicated that the Philos plate group experienced greater intraoperative blood loss, longer operative times, and higher postoperative VAS scores compared to the Multiloc group, suggesting a potentially significant impact on surrounding soft tissues and vascular supply. Additionally, the Philos plate group exhibited a higher incidence of postoperative complications, including screw cutout and humeral head necrosis, likely related to challenges in screw positioning and achieving precise intraoperative fracture reduction (14, 15). One potential factor contributing to the higher complication rate in the PHILOS group was the lack of intramedullary augmentation beneath the humeral head. In elderly patients with osteoporosis, structural support using autologous iliac crest bone grafts or synthetic bone substitutes may help to prevent humeral head collapse, necrosis, and hardware cut-out. However, in our practice, such augmentation was not routinely performed due to the increased surgical time, invasiveness, and donor site complications associated with bone graft harvesting. The decision to forgo augmentation was made based on intraoperative assessment of fracture stability and bone integrity. Nonetheless, this may represent a limitation in our treatment strategy, and future studies should further explore the role of intramedullary augmentation in optimizing outcomes for elderly patients undergoing plate fixation. To mitigate these risks, surgeons must accurately assess fracture fragments alignment during anatomical reduction and optimize screw length and orientation to prevent postoperative cut-out or loosening (16–18), which are more likely to occur with inexperienced surgeons. Recommendations include the use of precise fluoroscopic guidance to confirm the position and angle of each screw during surgery. Additionally, minimizing soft tissue dissection around the fracture site is essential to preserve humeral head vascularity. Furthermore, implementing individualized rehabilitation plans postoperatively is required (19–21).

Reverse shoulder arthroplasty (RSA) is recognized as an effective treatment option for elderly patients with complex proximal humeral fractures, particularly in cases involving irreparable rotator cuff tears or severe comminution. However, it was not employed in the present cohort. All patients included in this study had either an intact or reparable rotator cuff and adequate proximal humeral bone stock, making them suitable candidates for internal fixation. Furthermore, RSA is associated with higher costs, an increased risk of prosthesis-related complications, and prolonged rehabilitation, especially in resource-limited healthcare settings. At our institution, motion-preserving and joint-conserving surgical techniques are preferred as the first-line approach when stable fixation is feasible. Nevertheless, RSA remains a valuable alternative in selected patients and should be considered when internal fixation is unlikely to yield satisfactory outcomes.

MultiLoc intramedullary nail fixation is a minimally invasive technique offering high stability. Its unique multiplanar locking design enhances humeral head fixation and reduces the risk of fracture redisplacement (22–24). In this study, the Multiloc group demonstrated shorter incision lengths, lower intraoperative blood loss, and superior postoperative shoulder function than the Philos group. These findings suggest that the Multiloc method better preserves surrounding soft tissues and vascular supply, facilitating early postoperative recovery. Furthermore, the Multiloc group had fewer postoperative complications, highlighting its superiority in treating older patients with osteoporosis. The success of Multiloc intramedullary nail fixation depends on the precise selection of the entry point and the reaming technique. Postoperative complications such as proximal humeral refracture may result from inadequate mastery of reaming techniques (25, 26). To optimize surgical outcomes, ensuring precise fluoroscopic guidance during reaming is critical to prevent deviation from the fracture site, carefully position locking screws for stable multiplanar support that minimizes fracture fragment displacement, and to emphasize pain-free rehabilitation postoperatively, while avoiding early weight-bearing to prevent fixation failure (27, 28).

This study also revealed that the surgical method significantly influences early shoulder function recovery. The minimally invasive nature of Multiloc intramedullary nail fixation minimizes damage to the rotator cuff and surrounding soft tissues, thereby promoting superior postoperative recovery of shoulder function. Conversely, although Philos plate fixation offers excellent biomechanical stability in certain cases, the extensive soft tissue dissection required may increase postoperative pain and complications, thereby hindering early functional recovery, particularly in older patients with osteoporosis (29, 30). The Constant-Murley scores in this study demonstrated a significantly better functional recovery in the Multiloc group compared to the PHILOS group at 1, 3, 6, and 12 months postoperatively. This may be attributed to the minimally invasive nature of intramedullary nailing, which reduces soft tissue disruption and preserves rotator cuff integrity. Early initiation of functional exercises, facilitated by the biomechanical stability of the Multiloc construct, may have further contributed to improved shoulder mobility and strength in the early and mid-term phases of recovery. In contrast, although the PHILOS plate provides strong angular stability, the relatively extensive soft tissue exposure and the subacromial impingement potential may delay early mobilization and cause postoperative stiffness, which can negatively affect functional outcomes. These findings are consistent with previous studies reporting faster and more complete functional recovery in patients treated with intramedullary nails, especially in osteoporotic elderly populations.

In addition to functional outcomes, fracture healing was also evaluated and compared between the two groups. The Multiloc group exhibited a significantly shorter mean time to radiographic union (14.2 ± 2.1 weeks) than the PHILOS group (16.8 ± 2.6 weeks), with higher union rates observed at the 3-month follow-up (92.5% vs. 84.0%). This difference may reflect the biological and mechanical advantages of intramedullary fixation, which maintains alignment with minimal periosteal disruption and preserves the fracture hematoma—factors known to facilitate bone healing. Conversely, the longer healing time in the PHILOS group may be partially attributed to the more invasive surgical approach, which requires extensive soft tissue dissection and may impair local vascularity. Although all fractures eventually achieved union in both groups, the faster healing observed with the Multiloc nail could allow for earlier functional recovery and reduced complication risk, particularly in elderly osteoporotic patients who are more susceptible to delayed union.

Shoulder stiffness is a recognized complication following prolonged immobilization, particularly in elderly patients with diabetes. In our protocol, despite sling immobilization for six weeks, early initiation of passive and active-assisted ROM exercises helped minimize this risk. Patients with diabetes received individualized physiotherapy and close follow-up to detect and manage early signs of frozen shoulder. As a result, the incidence of clinically significant postoperative stiffness was low and did not differ substantially between groups. Nonetheless, future protocols may consider shortening sling duration or incorporating earlier aggressive mobilization in select high-risk individuals.

This study is limited by its small sample size and retrospective, single-center design, which may introduce selection bias. Additionally, variations in the surgeon expertise and patient adherence to postoperative rehabilitation protocol may have influenced the outcomes. Future studies should involve larger, multicenter, prospective, and randomized controlled trials to validate these findings and explore the efficacy of alternative surgical methods for treating complex proximal humeral fractures in elderly patients.

Multiloc intramedullary nail fixation demonstrated certain advantages in managing complex osteoporotic fractures in older patients, particularly in terms of functional recovery and complication reduction. In clinical practice, selecting the appropriate surgical method should be based on a comprehensive evaluation of individual patient characteristics and fracture type to achieve optimal treatment outcomes.

## Data Availability

The raw data supporting the conclusions of this article will be made available by the authors, without undue reservation.
